# A specific FMNL2 isoform is up-regulated in invasive cells

**DOI:** 10.1186/s12860-016-0110-z

**Published:** 2016-08-30

**Authors:** Christine Péladeau, Allan Heibein, Melissa T. Maltez, Sarah J. Copeland, John W. Copeland

**Affiliations:** Department of Cellular and Molecular Medicine, Faculty of Medicine, University of Ottawa, 451 Smyth Road, Ottawa, ON K1H 8M5 Canada

**Keywords:** Formins, Actin, Invasion, Metastasis, FMNL2, Melanoma, Colorectal cancer

## Abstract

**Background:**

Formins are a highly conserved family of cytoskeletal remodeling proteins. A growing body of evidence suggests that formins play key roles in the progression and spread of a variety of cancers. There are 15 human formin proteins and of these the Diaphanous-Related Formins (DRFs) are the best characterized. Included in the DRFs are the Formin-Like proteins, FMNL1, 2 & 3, each of which have been strongly implicated in driving tumorigenesis and metastasis of specific tumors. In particular, increased FMNL2 expression correlates with increased invasiveness of colorectal cancer (CRC) in vivo and for a variety of CRC cell-lines in vitro. FMNL2 expression is also required for invasive cell motility in other cancer cell-lines. There are multiple alternatively spliced isoforms of FMNL2 and it is predicted that the encoded proteins will differ in their regulation, subcellular localization and in their ability to regulate cytoskeletal dynamics.

**Results:**

Using RT-PCR we identified four FMNL2 isoforms expressed in CRC and melanoma cell-lines. We find that a previously uncharacterized FMNL2 isoform is predominantly expressed in a variety of melanoma and CRC cell lines; this isoform is also more effective in driving 3D motility. Building on previous reports, we also show that FMNL2 is required for invasion in A375 and WM266.4 melanoma cells.

**Conclusions:**

Taken together, these results suggest that FMNL2 is likely to be generally required in melanoma cells for invasion, that a specific isoform of FMNL2 is up-regulated in invasive CRC and melanoma cells and this isoform is the most effective at facilitating invasion.

## Background

Metastasis initiates with the migration of individual cells, or cellular collectives, from the tumor into the surrounding stroma [1]. Individually invading tumor cells use either a mesenchymal or amoeboid mode of 3-D migration. In this context, mesenchymal invasion requires low actomyosin contractility, greater cell-substrate attachment, and proteolytic digestion of the extracellular matrix. In contrast, amoeboid invasion requires high contractility, low adhesion and is protease-independent [2, 3]. A specific cell-type utilizes a specific mode of invasion, however, cells will switch from mesenchymal to amoeboid migration when mesenchymal motility is inhibited [4]. In vivo, amoeboid invasion is very rapid and is used by breast cancer cells to invade the stroma [5]. In vitro, a variety of colorectal and melanoma cancer cell lines adopt this mode of migration [3, 6]. Both 2D and 3D cell motility is driven by the coordinated regulation of actin dynamics and is dependent upon a variety of actin remodeling proteins.

Formin homology proteins are a highly conserved family of cytoskeletal remodeling proteins distinguished by the presence of two functional domains, formin homology 1 (FH1) and FH2. FH1 is proline-rich and a ligand for the small actin-binding protein profilin [7]. FH2 directly nucleates actin polymerization, inducing the formation of long, unbranched actin filaments (F-actin); some FH2 domains also bind and bundle F-actin [8–11]. FH2 is a dual purpose domain that is also able to regulate microtubule (MT) stabilization [12–14].

Given their dramatic effects on cytoskeletal dynamics, it is not surprising that a number of formins have been shown to play a role in metastasis and invasion [1]. In particular, the FMNL sub-group of Diaphanous-Related Formins have each been shown to be required for migration and invasion by transformed cells. FMNL1 promotes proliferation and motility of leukemia cells [15–17]. FMNL3 is required for invasion in PC3 prostate cancer cells and down-regulation of FMNL3 expression is associated with suppression of metastasis [18–21]. Increased FMNL2 expression correlates with increased invasiveness in CRC cell-lines [22–24] and FMNL2 is consistently highly expressed across the NCI60 panel of melanoma cell-lines [25]. In patient samples increased FMNL2 expression also correlates directly with increased CRC metastasis [23, 24]. FMNL2 is required in both MDA-MB-435 for amoeboid cell invasion in vitro and B16-F1 melanoma cells where it cooperates with the Arp2/3 complex for lamellipodial extension [6, 26]. More recently, FMNL2 has been shown to participate in the regulation of cell-cell and cell-substrate adhesions [27, 28].

The ability of FMNL2 to govern cytoskeletal dynamics is regulated by an autoinhibitory interaction between its C-terminal Diaphanous Autoregulatory Domain (DAD) and N-terminal Diaphanous Inhibitory Domain (DID) [11]. Inhibition is relieved by binding of either active RhoC or cdc42 to the FMNL2 GTPase Binding Domain (GBD) [6, 26]. Deletion of either DID or DAD is sufficient to render FMNL2 constitutively active [11]. As with other formins, constitutively active derivatives of FMNL2 are able to induce F-actin accumulation and MT acetylation in vivo [11, 12] as well as bind and bundle actin filaments and accelerate F-actin polymerization in vitro [11, 26]. F-actin bundling by FMNL2 is dependent on FH2 and an actin-binding WH2 motif immediately C-terminal to the FH2 domain [11]. The FMNL2 protein is targeted to the plasma membrane by N-myristoylation at Gly2 [29], although there are conflicting reports as to whether or not this modification is required for FMNL2-dependent cell migration [22, 26].

Many formin genes undergo alternative splicing of their mRNA and the resulting isoforms have significant impact on the regulation, localization and function of the encoded proteins [30–34]. Similarly, up-regulation of a specific splice form of the cytoskeletal remodeling proteins Mena and palladin potentiates the invasive phenotype of transformed cells [35–38]. There are at least 14 predicted alternative splice forms of FMNL2 affecting regions that are likely to impact on the activity, regulation and subcellular localization of the encoded proteins. We show here that there are at least four of the predicted FMNL2 isoforms expressed in CRC and melanoma cells and that a novel isoform is preferentially up-regulated in invasive cells. We also show that invasion by A375 and WM266.4 melanoma cells is FMNL2-dependent suggesting that FMNL2 might be generally required for 3D invasion in this cell-type. All four FMNL2 isoforms are able to rescue invasion in FMNL2-depleted A375 cells, but the “invasive” isoform is significantly better than the other three.

## Methods

### Cell culture

U2OS osteosarcoma human cells were a gift from Dr. Laura Trinkle-Mulcahy and were grown in DMEM 10 % FBS at 37 °C, in 5 % CO_2_. All other cell-lines were obtained from the American Type Culture Collection (ATCC) and were maintained as recommended by the supplier.

### Plasmids

The FMNL2 isoforms described in this study correspond to the following accession numbers. ITM: NP_443.137.2, YHY: Q96PY5, PMR: XP_005246322, TQS: XP_005246320.1. Full length (FL) FMNL2 isoform cDNA were assembled in a step-wise fashion into the pEF-plink2-mCherry vector. Briefly, the alternative 3′ ends of each FMNL2 isoform was amplified by polymerase chain reaction (PCR) from cDNA generated from SW620 cell mRNA. The conserved 5′ portion of the FMNL2 cDNA was amplified from KIAA1902 (Kazusa Project, Japan). FMNL2 FH1 + FH2 derivatives were subcloned into pEF.NBRSS from the full-length constructs. For rescue, FMNL2 full-length isoforms were cloned into pLVX-IRES-mCherry lentivirus vector. All PCR reactions were performed using standard techniques and the Phusion High Fidelity DNA Polymerase (Thermo scientific).

### Serum Response Factor (SRF) assay

SRF reporter gene assays were performed as described previously (Copeland and Treisman [39]). Briefly, NIH 3T3 fibroblasts were seeded on 6-well plates at 125 000 cells/well 1 day prior to transfection. The cells were transfected using polyethylenimine transfection reagent (PEI) with the indicated expression plasmids (0.1ug) and the reporter constructs p3D.A.Luc (50 ng/well) and pMLVLacZ (250 ng/well). After 5 h, the cells were placed in low serum medium (0.5 % FBS in DMEM). Cells were harvested the next day and lysed in 1x Reporter Lysis buffer; luciferase assays were performed according to the supplied protocol (Promega) and read in an LMAXII Luminometer (Molecular Devices). The activation of luciferase was standardized to an SRF-VP16 control. A β-galactosidase (β-gal) assay is performed in parallel as a transfection efficiency control. The cell lysates are also subjected to a sodium dodecyl sulfate polyacrylamide gel (SDS-PAGE) and immunoblotted to detect FMNL2 expression levels.

### RNA extraction, RT-PCR, and qPCR

The indicated cell-lines were grown to 70–80 % confluence in 10 cm dishes. Total RNA was harvested using the RNeasy mini kit (Qiagen). Total RNA concentration was determined by reading the OD_280_. RNA quality was assessed on denaturing formaldehyde/agarose MOPS gel and visualization of distinct 18S and 28S ribosomal subunits. cDNA were synthesized using MuLV reverse transcriptase and the GeneAmp RNA PCR kit (Applied Biosystems). PAW109 RNA serves as a positive RT-PCR control and no RNA (ddH_2_0) served as negative control. Characterization of the FMNL2 isoforms in A375, SW620 and SW480 cancer cell lines was accomplished by performing a PCR reaction [2 mM MgCL2 solution, 1X PCR buffer I, ddH_2_O, 2.5U/100 μL AmpliTaq DNA Polymerase, 20 μL purified cancer cell cDNA and 0.15 μM of both forward and reverse FMNL2 or control specific primers]. PCR products were separated on 1 % agarose gels. Amplified bands were isolated with Gen Elute Gel extraction kit (Sigma) and directly sequenced. These sequences were used to design primers for qPCR.

Real time PCR analysis was performed to determine absolute expression levels of FMNL2 isoforms using the following primers. ITM forward 5′-GCCATTGAAGATATTATCACAGATC-3′, rev 5′-AACTTGCGTTCTGTTAATGGTG-3′, amplicon 117 bp; YHY 5′-CTGAAGACTGTGCCCTTTACTGCT-3′, 5′-CCTGTTCTCACTGAGGAATACCATTAC-3′, amplicon 87 bp; PMR 5′-CATTGAAGATATTATCACAGCCTTA-3′, 5′-GAGGATCTTAGAAACCAACCATA-3′, amplicon 87 bp; TQS 5′-GATATTATCACAGCCTTAAAGAAGAAT-3′, 5′-TGGTGGAGGATACACAGAGCT-3′, amplicon 106 bp; pan-FMNL2 5′-GCTCCTCCCTTAGCACCT-3′, 5′-GCCAATCAAGACGAAGTTCAGA-3′, amplicon 127 bp; β-actin 5′-GCACCACACCTTCTACAATGAG-3′, 5′-GACCCAGATCATGTTTGAGACC-3′, amplicon 122 bp. Standard curves were established using FMNL2 FH1 + FH2 + C templates corresponding to each isoform. A logarithmic series of template dilutions were generated (10^2^, 10^3^, 10^4^, 10^5^, 10^6^ copies per tube). A standard curve for β-actin template was also generated as a control. The experimental design included standards, no template controls and unknowns. Standards contained: 5 μL FMNL2 isoform template, 5 μL of specific FMNL2 isoform primer (final concentration: 300 ng/well/primer) and 10 μL of the SYBR Green PCR Premix (QuantiTect; QIAGEN) used according to the manufacturer’s instructions. Unknown reactions consisted of 5 μL cDNA (harvested from cancer cell lines) (0.05 μg/tube), 5 μL primers and 10 μL SYBR Green qPCR Premix. No template control reactions were 5 μL of primers, 10 μL of SYBR Green mix and 5 μL ddH_2_O. 5-carboxy-X-Rhodamine (ROX) is also included in the SYBR Green qPCR Premix and serves as a passive loading reference dye. The qPCR reactions were run on an Mx 3005P qPCR instrument (Stratagene). Standard curves for each isoform were used to determine the relationship between the fluorescent signal (the CT value) and absolute copy number of each FMNL2 isoforms in each sample.

### Immunofluorescence

NIH-3T3 fibroblast cells were seeded at a density of 125 000 cells/well in 6-well plates containing acid-washed coverslips and 10 % DBS DMEM medium. The cells were transfected using PEI (1 mg/mL) as above. 24 h later, the cells were fixed with 4 % paraformaldehyde (PFA) in PBS for 15 min, washed three times with 1x PBS (5 min) and permeabilized for 30 min with 0.3 % Triton X-100, 10 % horse serum in 1XPBS. Fixed cells were incubated at room temperature with primary antibodies in 0.03 % Triton X-100 plus 5 % horse serum 1X PBS for 1 h. Cells were washed three times in 1X PBS and incubated with the appropriate secondary antibody and Fluorescein-Phalloidin (1:100, Molecular Probes) in 0.03 % Triton X-100 plus 5 % horse serum 1X PBS at room temperature for 1 h. The cells were washed three times in 1X PBS and once in ddH2O and mounted on slides with Vectashield mounting medium with DAPI (Vector Laboratories). Images were captured on a Zeiss Axio Imager Z1 fluorescence microscope, using a 63X Plan Apochromat objective and an AxioCam HRm camera. Optical sections were obtained with the apotome2. Images were processed using Axiovision software and figures assembled using Adobe Photoshop.

Filopodia formation in transfected cells was assessed visually by immunofluorescence. Transfected cells expressing myr-mCherryFP or FMNL2-mCherry were identified by virtue of the mCherryFP tag and F-actin visualized by fluorescein-phalloidin as above. Cells with an obvious over-proliferation of filopodia (>25 filopodia/cell) compared to either non-transfected neighbours or myr-mCherryFP expressing control cells were counted and the result expressed as a percent of transfected cells.

### Western blotting

Cells were grown to 70–80 % confluence in 10 cm dishes, washed with 1XPBS and harvested in 1xSDS buffer. Lysates were subjected to SDS-PAGE and immunoblotted with the indicated antibodies. Chemiluminescence was used for detection using the western HRP substrate reagent (Millipore) and visualized on a GE Image Quant LAS4010 Imaging System. Blots were stripped in 0.1 M Glycine, 0.5 % SDS (pH2.5) for 1 h. Blots were re-blocked and probed with mouse-anti-α-tubulin (clone DM1A, T9026, Sigma) to assess loading. Chemiluminescence and visualization is performed as described previously. Pan-FMNL2 antiserum was raised in chicken (Cedarlane) using the isolated FMNL2-FH2 protein (codons 599–1045) expressed in *E.coli* and purified as previously described [11]. All FMNL2 antisera were affinity purified using standard protocols [40]. Affinity purified anti-FMNL3 antibody was described previously [41].

### FMNL2 siRNA

A375 or WM266.4 melanoma cells were seeded in six well plates or 3.5 cm dishes (Corning) at a density of 125 000 cells/well. The following day, cells were transfected (DharmaFECT #1, Thermo Scientific) with control or FMNL2 siRNA duplexes (TriFECTa Dicer-Substrate RNAi Kit, Integrated DNA Technologies) as directed by the manufacturer. The siRNA duplex targeted the 3′UTR of FMNL2 (5′-CCUGUUCAGAUUAAUCAAAGCAATA-3′). A non-specific universal negative control duplex (Integrated DNA Technologies) was used for all siRNA knockdown experiments. This control duplex does not recognize any sequences in human, mouse or rat transcriptomes (5′-CGUUAAUCGCGUAUAAUAAGAGUAT-3′). Following transfection, cells were incubated at 37 °C (5 % CO_2_) for 48 h. A fluorescent TYE 563 DS control was used to verify transfection efficiency. After 48 h, cells are harvested and the lysates subjected to immunoblotting to detect FMNL2 expression levels.

### 2-D migration assay

A375 melanoma cells were seeded in six well plates or 3.5 cm petri dish (Corning) at a density of 125,000 cells/well. The following day, the cells were transfected with siRNA; after 48 h 100,000 A375 cells were added to each chamber of an ibidi wound insert in a 3.5 cm petri dish (Ibidi). The outside of the insert was filled with 1.5 ml of DMEM 10 % FBS. In parallel, cells were also seeded in duplicate to assess knockdown efficiency by immunobloting. The next day, the insert was removed to generate the wound and the plate was gently washed with 10 % FBS DMEM to remove any floating cells. Wound closure was monitored for 48 h by live imaging on a Zeiss Axiovert 200 microscope (10x objective, phase 1) in a controlled environment (5 % CO2, 37 °C). The percent wound closure was calculated by measuring the distance of the gap at three points using Northern Eclipse Software (NES, Empix Imaging, Mississauga, Ontario, Canada).

### Virus production and transduction

FMNL2 cDNA were cloned into the lentiviral vector pLVX-IRES-mCherry for virus production. Briefly, 10 plates (15 cm) of 293 T cells at 70 % confluence were transfected with 96.85 μg of the FMNL2 pLVX-IRES-mCherry construct, 53.95 μg of the envelop plasmid (pMD2G coding for VSV-G envelope), 99.15 μg of the packaging plasmid psPAX2 using PEI. Virus was collected from the medium supernatant every day for the next 48 h. The virus was concentrated and titrated to determine the multiplicity of infection (MOI). For rescue experiments, A375 melanoma cells were seeded at a density of 125 000 cells/well, in a six well plate or in a 3.5 cm petri dish with a coverslip. The next day, the cells were transfected with siRNAs and incubated for 24 h before infection with the FMNL2 expressing lentiviral vectors using a *multiplicity of infection (MOI*) of 10. The cells were left for another 24 h before seeding for an invasion assay. Efficiency of knockdown and re-expression of FMNL2 was assessed by immunoblotting and immunofluorescence on parallel samples.

### Transwell invasion assay

6.5 mm/8 μm transwell inserts (Costar #3422) were coated with 100 μL of 1:1 DMEM:growth factor reduced Matrigel (#356230) and allowed to polymerize for 1 h at 37 °C. The polymerized transwells were flipped upside down and the underside coated with 50 μL of a 1:20 matrigel: DMEM solution to facilitate cell adhesion and placed back into the 37 °C incubator for 1 h. 7000 A375 cells in 70 μL of DMEM (0.5 % FBS) were seeded on the underside of the inserts and allowed to adhere for 3 h at 37 °C. The inserts were then flipped into a 24 well plate containing 600 μL of DMEM (0.5 % FBS) on the bottom. 200 μL of 20 % FBS DMEM was carefully added to the top of the insert. Cells were left to invade through the matrigel at 37 °C (5 % CO_2_) for 72 h. The cells were then fixed with CSK buffer (containing 8 % paraformaldehyde) for 30 min, washed with PBS and then permeabilized with 0.3 % Triton X-100 for another 30 min. The cells were stained with 50 μg/mL of Propidium Iodide overnight at 4 °C. The transwells were then gently washed in PBS, rinsed in ddH_2_O, and then placed onto a 35 mm Mat-Tek coverslip culture dish with 15 μL Vectashield (Vector Labs). Cells were imaged on a Zeiss LSM.Pascal using a 40x NA1.30 oil objective. 100 optical sections were taken (50 slices before the transwell membrane and 50 slices into the matrigel) with the Z step set at Nyquist sampling frequency (~0.5 μM/slice) and total number of cells counted in each section to determine invasion frequency.

## Results

**Fig. 1:**
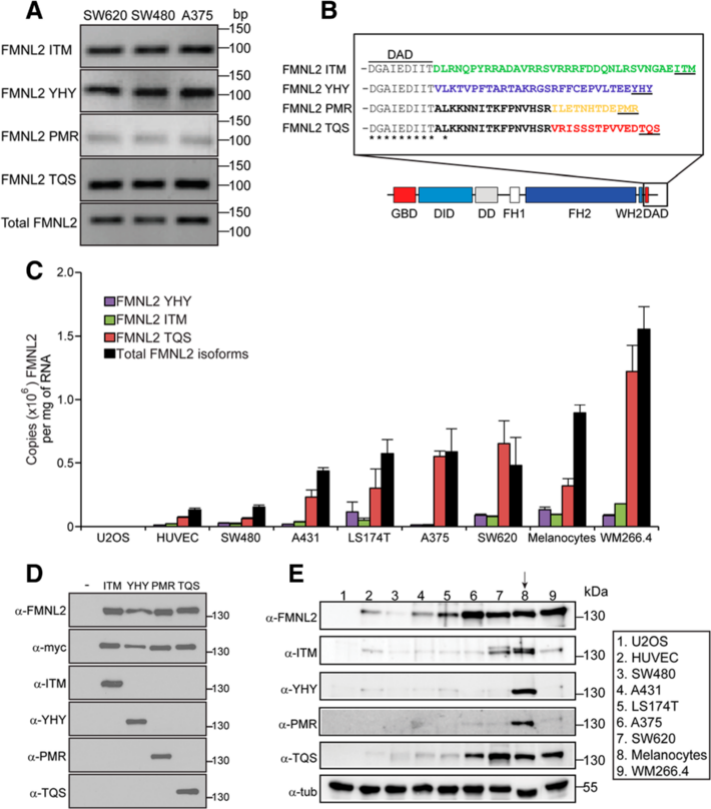
A specific FMNL2 isoform is over-represented in invasive cells. **a** RT-PCR was performed on total RNA harvested from SW480, SW620 and A375 cells. Specific primers were used to demonstrate the presence of four alternatively spliced FMNL2 isoforms identified in an initial RT-PCR analysis (see Results). **b** The four alternatively spliced mRNA are predicted to encode proteins differing only at amino acid residues C-terminal to the DAD domain. **c** qPCR was used to assess the levels of expression of each isoform in comparison to total FMNL2 in the indicated colorectal and melanoma cell-lines as well as in non-transformed primary endothelial cells and melanocytes. The TQS isoform was predominant in all the invasive cell-lines that were assessed. We were unable to obtain reliable qPCR data for the PMR isoform. **d** Isoform specific antibodies were raised against peptides corresponding to the unique domain of each C-terminal tail. Full-length derivatives of the indicated FMNL2 isoforms bearing N-terminal myc epitope tags were expressed in U2OS cells by transient transfection and the resulting cell lysates were immunoblotted with the indicated antibodies. Total FMNL2 protein was detected using an antibody directed against FMNL2 codons 599–1045. Equivalent samples were loaded on a separate gel and probed with α-myc antibodies. The α-FMNL2 blot was stripped and re-probed with α-TQS antibody; the α-myc blot was stripped and re-probed with α-PMR antibody. Equivalent samples were loaded on separate gels to detect ITM and YHY **e** Immunoblot analysis using antibodies directed against the C-terminal tails of each of the predicted FMNL2 isoforms confirms the qPCR analysis. Total FMNL2 protein was detected using pan FMNL2 antibody shown in 1D. Isoform specific antibodies were raised against peptides corresponding to the unique domain of each C-terminal tail. Equivalent samples were loaded on separate gels to eliminate concerns regarding incomplete stripping. The pan-FMNL2 blot was stripped and re-probed with anti α-tubulin for a loading control

There are 14 human FMNL2 isoforms listed in the NCBI protein database at present. These isoforms are generated by alternative splicing of their mRNA and the exons affected encode both functional and regulatory domains in the FMNL2 protein. Therefore we wished to determine which FMNL2 isoforms are expressed in cell-types relevant to the study of the role of FMNL2 in invasion and metastasis. A series of overlapping primer pairs was designed to span the entire FMNL2 coding sequence with attention paid to regions bridging the predicted alternative splice sites. These primers were used to amplify individual sections of the entire FMNL2 coding sequence using cDNA prepared from SW480 to SW620 colorectal cancer, and A375 melanoma, cell-lines. The resulting amplified fragments were gel-purified and sequenced directly. From this analysis only four FMNL2 alternatively spliced isoforms were detected and these varied only at the 3′ end of the coding sequence (see Experimental Procedures for accession numbers). Based on sequence analysis, additional primer pairs were designed to confirm these results directly and to amplify these sequences specifically (Fig. 1a). The proteins encoded by the alternatively spliced mRNA differ only in the region C-terminal to the autoregulatory DAD domain and are designated ITM, PMR, YHY, and TQS for the final three C-terminal amino acid residues of each protein (Fig. 1b). It should be noted that additional alternatively spliced versions of FMNL2 are listed in the NCBI database, however, all of these splicing events are predicted to affect other regions of the coding sequence, that is, there are no additional 3′ splice variants predicted that encode additional alternative C-terminal tails. No other splice variants were detected in our assays.

In our initial RT-PCR analysis we noted apparent variations in the relative abundance of the different FMNL2 splice forms (Fig. 1a). Therefore we wished to determine the expression levels of each isoform in a panel of relevant cell-lines chosen either for utilizing an amoeboid mode of migration, or for their tissue of origin (CRC and melanoma). Primary HUVECs, primary melanocytes, A431 carcinoma and U2OS osteosarcoma cells were also included for comparison. Primers unique to each alternatively spliced isoform were designed for qPCR analysis and amplification efficiency was calibrated using the relevant cloned cDNA. Validated primer sets were obtained for the linear amplification of total FMNL2, ITM, YHY and TQS, however, we were unable to generate primers for the specific linear amplification of PMR. The cloned cDNA were also used to generate standard curves that allowed the derivation of absolute copy number for each isoform in the various cell-lines. Consistent with previous reports [23], we confirmed that FMNL2 expression is elevated in the metastatic SW620 cell-line when compared to parental SW480 cells (Fig. 1c). We also detected similar elevated levels of expression in LS174T CRC cells, as well as A375 and WM266.4 melanoma cells (Fig. 1c). We were unable to detect FMNL2 mRNA in U2OS cells and only low levels of FMNL2 mRNA in primary HUVECs. Surprisingly, the total level of FMNL2 expression in primary melanocytes was similar to that in the melanoma cell-lines. Specific, individual measurement of the separate FMNL2 isoforms indicated that TQS was essentially the sole isoform expressed in A375, WM266.4 and SW620 cells and the major isoform in LS174T and A431 cells. In primary melanocytes, however, TQS accounts for less than 50 % of total FMNL2 mRNA. We were unable to assess levels of PMR expression in this assay. Nevertheless, a comparison of total FMNL2 with the sum of the individual isoforms suggests that, with the notable exception of primary melanocytes, PMR does not contribute significantly to total FMNL2 mRNA levels.

We next wished to confirm the results of our qPCR assays at the protein level. Total FMNL2 levels were assessed using a pan-FMNL2 antibody recognizing an invariant region in the N-terminus. We also generated polyclonal antibodies raised against peptides corresponding to the unique C-terminal tails of each of the four FMNL2 isoforms (Fig. 1d). These antibodies were affinity purified and used for immunoblot analysis of lysates from the panel of cell-lines used in Fig. 1c. The pan-FMNL2 immunoblot (Fig. 1e, top panel) largely confirmed the qPCR results, with highest levels of FMNL2 expression in primary melanocytes and metastatic melanoma (A375 and WM266.4) and CRC (LS174T and SW620) cell-lines. FMNL2 protein was not detected in U2OS cell lysates. The isoform specific immunoblots also showed that only primary melanocytes express a mix of the four FMNL2 isoforms; significant levels of YHY and PMR are not detected in the other cell-lines and only low levels of ITM are detected across this panel. In contrast, the TQS specific immunoblot shows that it is the major isoform expressed in A375, SW620 and WM266.4 cell-lines.

**Fig. 2:**
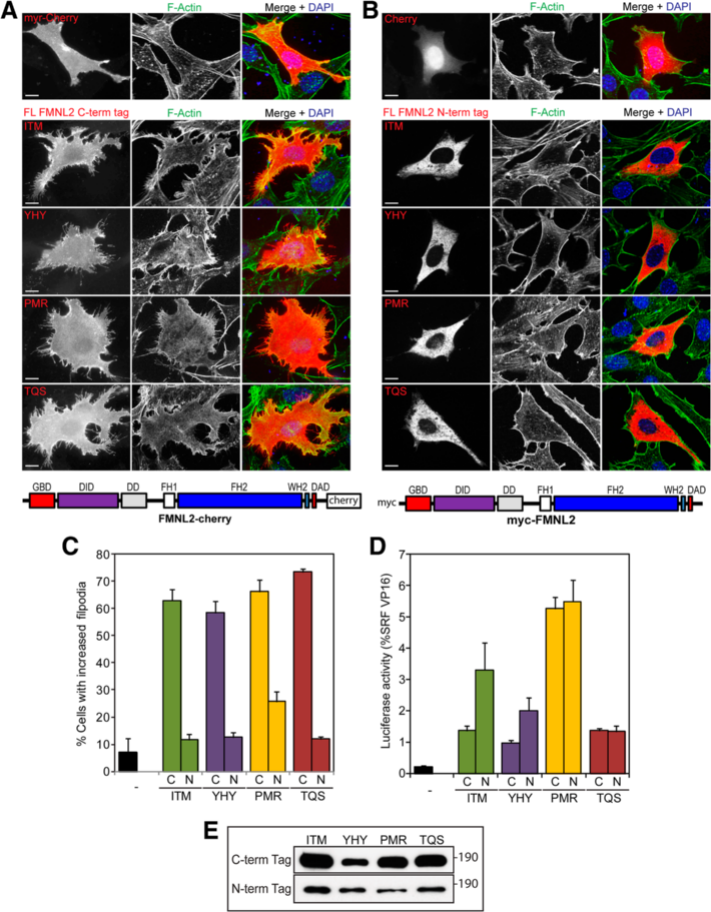
FMNL2 expression induces filopodia formation. **a** C-terminally tagged full-length derivatives of each FMNL2 isoform were expressed in NIH 3T3 fibroblasts by transient transfection. F-actin (*green*) was detected with phalloidin and FMNL2 expression (*red*) was detected by virtue of the C-terminal mCherry tag. Nuclei were detected with DAPI (*blue*) in merged image. FMNL2 expression induced extensive filopodia formation in comparison to untransfected cells or cells expressing mCherryFP with an N-terminal myristoylation motif (*top panel*). **b** N-terminally tagged full-length derivatives of each of the FMNL2 isoforms were expressed in NIH 3T3 cells by transient transfection. F-actin (*green*) was detected with phalloidin and FMNL2 expression (*red*) was detected by virtue of the N-terminal myc tag. Expression of N-terminally tagged FMNL2 isoforms does not induce filopodia formation. mCherryFP was included as a control (*top panel*) **c** Quantification of data shown in (**a, b**). “C” indicates C-terminal mCherry tag, “N” indicates N-terminal myc tag. *N* = 3, >100 cells counted per sample, error bars = SEM. **d** NIH 3T3 fibroblasts were transiently transfected with an SRF luciferase reporter gene and the indicated full-length FMNL2 derivative. “C” indicates C-terminal mCherry tag, “N” indicates N-terminal myc tag. Reporter activation is expressed relative to an SRF-VP16 control fusion protein. *N* = 3, error bars = SEM. **e** Equivalent samples of transfected cell lysates were subjected to SDS-PAGE and immunoblotted. Expression of the indicated FMNL2 isoforms was detected with either anti-mCherry or anti-myc antibody

Amino acid residues C-terminal to the DAD motif are predicted to modulate formin autoregulation [9] and modify FH2 activity [42]. To compare activity of the FMNL2 isoforms, we generated full-length cDNAs encoding each of the four splice variants. FMNL proteins are predicted to be N-myristoylated [26, 29, 33] and this modification may play some role in targeting these proteins to the plasma membrane. To assess the effects of N-myristoylation we generated FMNL2 derivatives with either N-terminal myc epitope tags or C-terminal mCherryFP tags. These were expressed in NIH 3T3 cells by transient transfection and their subcellular localization and effects on cell morphology and the actin cytoskeleton were assessed by immunofluorescence (Fig. 2). The C-terminal tagged derivatives were recruited to the plasma membrane where they induced extensive filopodia formation (Fig. 2a, c). This was not the case with the N-terminal tagged proteins, which were distributed diffusely throughout the cytoplasm. Expression of N-terminal tagged versions of ITM, YHY or TQS did not induce filopodia formation above the background levels seen in control cells while expression of PMR was only sufficient to induce filopodia formation in a minority of cells (Fig. 2b, c). We also compared activity of N- and C-terminal tagged proteins using an SRF reporter gene assay. This reporter gene is activated by the MRTF/SRF transcription pathway in response to depletion of cellular pools of G-actin and is an indirect, sensitive and quantitative measure of changes in actin dynamics [39]. Neither N- nor C-terminal tagged full-length derivatives of the FMNL2 isoforms induced robust activation of the SRF reporter in this assay suggesting that, as expected, the full-length derivatives of each isoform are not constitutively active. Some differences, however, were apparent between the four isoforms, both in terms of basal activity and the effects of the position of the tag. PMR showed the most activity, inducing activation of the SRF reporter gene to similar levels above background for both N-and C-terminal tagged derivatives (Fig. 2d). In contrast, the C-terminally tagged full-length derivatives of ITM, YHY and TQS were largely inactive, while the N-terminal tag on ITM had a modest effect on its autoinhibition as well.

**Fig. 3:**
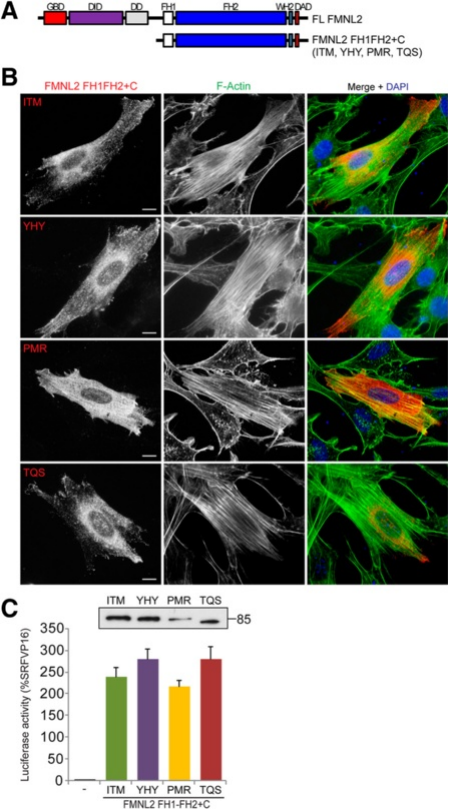
Constitutively active derivatives of FMNL2 induce stress fiber formation. **a** Schematic of FH1-FH2 + C containing derivatives of each FMNL2 isoform. **b** FH1-FH2 + C containing derivatives of FMNL2 were expressed in NIH 3T3 cells by transient transfection. Expression of each of the FMNL2 isoforms (*red*) was sufficient to induce stress fiber formation (F-actin, *green*). Nuclei were detected with DAPI in the merged image. **c** NIH 3T3 fibroblasts were transiently transfected with an SRF luciferase reporter gene and the indicated FMNL2 derivative. Reporter activation is expressed relative to an SRF-VP16 control fusion protein. *N* = 3, error bars = SEM. Equivalent samples of transfected cell lysates were subjected to SDS-PAGE and immunoblotted using an anti-myc antibody (*inset*)

The results of the SRF reporter gene assays suggest that the full-length FMNL2 isoforms are largely autoinhibited and that FMNL2-induced filopodia are likely a product of basal levels of FMNL2-induced F-actin formation on top of remodeling of pre-existing actin filaments. We therefore also wanted to compare the activity of constitutively active derivatives of the four isoforms. Deletion derivatives consisting of FH1, FH2 and the C-terminal tail (FH1FH2 + C) of each isoform were expressed in NIH 3T3 cells by transient transfection and the effects on cell morphology and actin polymerization were assessed by immunofluorescence (Fig. 3a, b). All four isoforms behaved similarly in this assay and induced a typical “formin” phenotype with extensive formation of thin stress fibers running from one end of the cell to the other. No obvious differences were apparent in the subcellular localization of each of these derivatives either. The SRF reporter gene assay was also used to compare the effects of each isoform on actin dynamics. Expression of the FH1FH2 + C derivative of each isoform was sufficient to induce robust SRF reporter gene activation consistent with previous results for ITM [11]. As with the formation of stress fibers, there was no obvious difference in the level of activation of the SRF reporter induced by expression of each isoform.

**Fig. 4:**
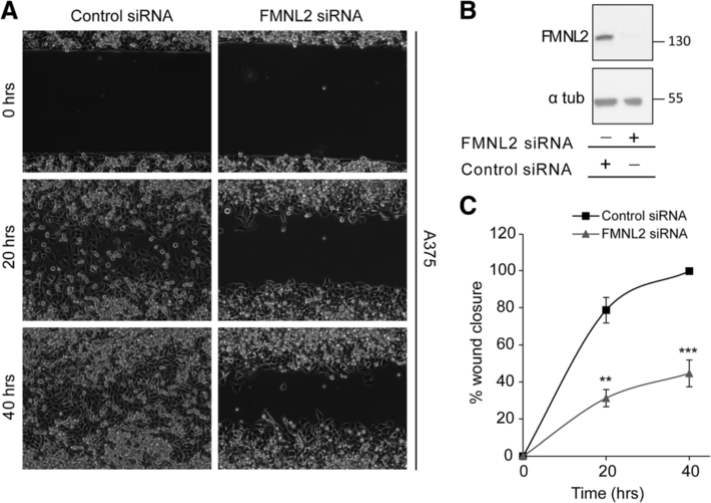
FMNL2 is required for 2-D migration in A375 melanoma cells. **a** A375 cells were transfected with either control siRNA duplex or duplexes targeting all FMNL2 isoforms. The transfected cells were grown to confluence in scratch wound assay chambers (see Experimental Procedures). Wound closure was followed at the indicated times by phase contrast live-cell microscopy. **b** Equivalent samples of cell lysates from (**a**) were subjected to SDS-PAGE and immunoblotted to detect FMNL2 expression with anti-FMNL2 antibody. The pan-FMNL2 blot was stripped and re-probed with anti α-tubulin as a loading control. **c** Quantification of the data shown in (**a**). The gap in the cellular monolayer is represented as % wound closure. *N* = 3, error bars = SEM. ** indicates *P* < 0.01 and *** indicates *P* < 0.001

Our preliminary analysis of the full-length FMNL2 isoforms suggested that there are some functional differences between them (Fig. 2c, d). We wished to determine if this extends to their effects on cell behavior. Previous work has suggested that FMNL2 is required for both 2D and 3D cell motility in specific cell-types [6, 26]. Therefore we wanted to determine if FMNL2 is required for motility in A375 cells which express the TQS isoform almost exclusively. We used siRNA duplexes targeted against FMNL2 to knockdown its expression in A375 cells and the level of depletion was assessed by immunoblotting with an anti-FMNL2 antibody (Fig. 4b); cells transfected with non-specific duplex were used as a control. FMNL2 expression was knocked down very efficiently and the effects of FMNL2 depletion on cell motility were assessed using a variation on the scratch wound assay. In this assay, cells were seeded in a culture insert composed of two chambers separated by a divider and allowed to grow to confluence. Removal of the insert creates a standard “wound” and migration into the wound is followed by live-cell imaging. Control cells extensively infiltrated the wound by 20 h and filled the wound by 40 h (Fig. 4a, c). In contrast, FMNL2-depleted cells made little progress into the wound even after 40 h (Fig. 4a, c). This suggests that FMNL2 plays a role in 2D migration in A375 cells and encouraged us to investigate if the same holds true for 3D invasion.

**Fig. 5:**
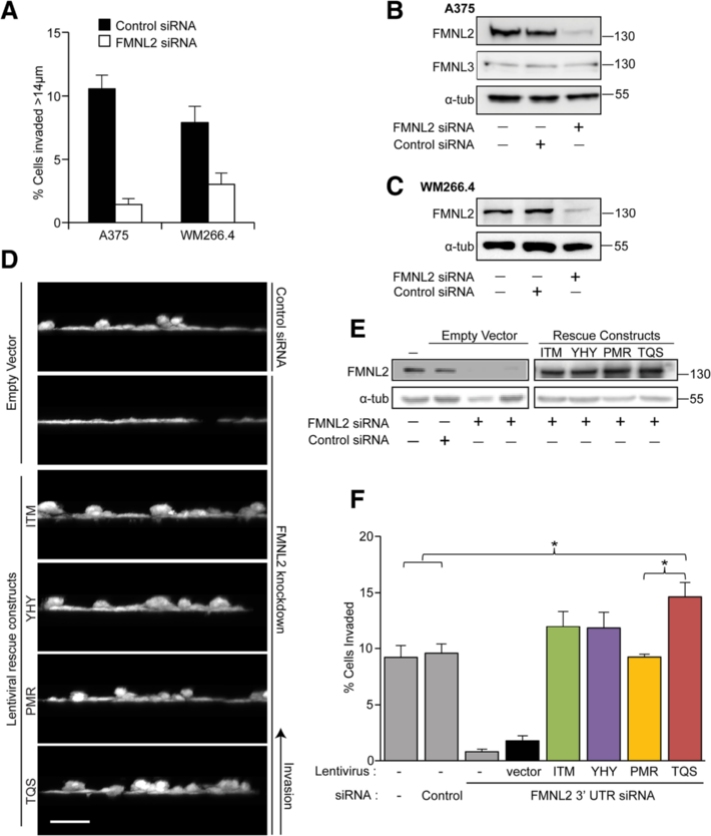
FMNL2 is required for invasion in A375 and WM266.4 melanoma cells. **a** A375 and WM266.4 melanoma cells were transfected with control or FMNL2-specific siRNA duplexes. Control and knockdown cells were then assayed for their ability to invade in vitro in an inverted Boyden chamber assay (Kitzing et al. [6]). FMNL2 knockdown inhibited invasion in both A375 and WM266.4 cells. **b** Equivalent samples of A375 cell lysates from (**a**) were subjected to SDS-PAGE and immunoblotted to detect FMNL2 and FMNL3. The pan-FMNL2 blot was stripped and re-probed with anti α-tubulin as a loading control. **c** Equivalent samples of WM266.4 cell lysates from (**a**) were subjected to SDS-PAGE and immunoblotted to detect FMNL2. The pan-FMNL2 blot was stripped and re-probed with anti α-tubulin as a loading control. **d** A375 cells were transfected with control and FMNL2-targeted siRNA duplexes and then infected with control lentiviral vector or lentiviral vectors expressing the indicated untagged full-length FMNL2 isoforms. The cells were then assayed for their ability to invade as in (**a**). Images show a representative Z-stack projection from the invasion assays. **e** Equivalent samples of cell lysates from (**d**) were subjected to SDS-PAGE and immunoblotted to detect FMNL2. α-tubulin was used as a loading control. **f** Quantification of data shown in (**d**). *N* = 3, error bars = SEM. *indicates *P* < 0.05 where noted. There was no significant difference between TQS and ITM or TQS and YHY

A375 and WM266.4 melanoma cells both invade using an amoeboid mode of 3D migration [3]. To determine if FMNL2 is required for 3D migration in these cells, we used a modified Boyden chamber assay to measure invasion [6]. In this assay cells are seeded on the underside of a transwell insert and their ability to invade upward into a matrigel matrix is assessed by confocal microscopy. siRNA duplexes were used to knockdown FMNL2 expression in both A375 and WM266.4 cells and FMNL2 was efficiently depleted in both cell-types as measured by immunoblotting (Fig. 5b, c). FMNL2 depletion did not affect expression levels of the related protein FMNL3 in A375 cells (Fig. 5b) and expression of FMNL1 was not detected (data not shown). The effect of FMNL2 depletion on the ability of A375 and WM266.4 cells to invade was compared to control cells transfected with non-specific siRNA duplexes. For both cell-types we found that FMNL2 depletion was sufficient to significantly inhibit invasion in the Transwell assay (Fig. 5a).

Having shown that FMNL2 is required for invasion in these cells, we wanted to compare the relative ability of each of the four FMNL2 isoforms to rescue invasion in knockdown cells. A375 cells were selected for this experiment as they were more invasive in our assay, FMNL2 was efficiently knocked down, and the effect of FMNL2 knockdown on invasion was more striking with these cells than with WM266.4 (Fig. 5a–c). Lentiviral rescue vectors were generated for ITM, YHY, PMR and TQS. This vector does not encode an epitope tag on the protein of interest, but does express mCherryFP as a separate protein via an IRES within the same mRNA. Empty vector expressing mCherry alone was used as a control. Using these vectors we were able to achieve nearly 100 % transduction efficiency in A375 cells. As before, transfection of control siRNA duplex had no effect on invasion while knockdown of FMNL2 strongly inhibits it. Efficiency of knockdown, and inhibition of invasion, is not affected by infection with the control lentiviral vector in FMNL2-depleted cells (Fig. 5d–f). In contrast, expression of each of the individual FMNL2 isoforms was sufficient to rescue invasion. It should be noted, however, that each isoform did not rescue to the same extent. PMR was the least efficient, TQS was the most efficient, while YHY and ITM fell in between. Indeed, the enhanced invasion observed with TQS was significantly greater than both the parental cell-line and PMR-rescued cells (although not significantly different from ITM or YHY). This suggests that there are unique functional differences between FMNL2 isoforms when it comes to driving invasive cell migration.

## Discussion

We provide here the initial description of two previously uncharacterized isoforms of FMNL2 (PMR and TQS) and show that at least four alternatively spliced FMNL2 isoforms are expressed in primary melanocytes. In contrast, the TQS isoform predominates in invasive melanoma and CRC cell-lines and we find that knockdown of FMNL2 expression in A375 and WM266.4 melanoma cells is sufficient to inhibit invasion in an in vitro assay. Taken with previous reports that FMNL2 is required for migration in mouse B16 melanoma cells and for invasion in MDA-MB-435 [6, 26], our results suggest that FMNL2 is likely to be generally required for invasion in melanoma cells. Like MDA-MB-435 cells, both A375 and WM 266.4 cells use an amoeboid mode of migration for 3D invasion and these results suggest that this mode of invasion might also generally require FMNL2 or related activity [19].

At least four FMNL2 isoforms (ITM, YHY, PMR and TQS) are expressed in the assessed cell-types at both the transcript and protein level. Although we cannot discount that additional isoforms may be expressed in other cell-types, we did not find any evidence for additional isoforms in our assays. In addition, the four unique C-terminal tails we describe are the only C-termini predicted for all FMNL2 isoforms currently listed in the NCBI database. Thus, the antiserum directed against each unique FMNL2 C-terminal peptide should account for all predicted isoforms of FMNL2. In primary melanocytes all four splice forms were readily detected at the protein level, however, in WMM266.4, A375 and SW620 cells TQS is by far the predominant isoform. This raises the intriguing possibility that TQS represents an “invasive isoform” of FMNL2 similar to previous descriptions of invasive isoforms of Mena [43] and palladin [35]. In support of this hypothesis we find that in FMNL2-depleted A375 cells, TQS is able to rescue invasion significantly better than PMR and to a significantly higher level than control cells. It should be noted that under the conditions of the rescue experiment, the levels of exogenous FMNL2 expression were considerably higher than the endogenous protein and it is likely that the system is saturated for FMNL2 activity. If that is the case, it would suggest that the observed effects represent the maximal potentiation of invasion by each isoform in these cells.

Increased FMNL2 expression is reported to directly correlate with increased invasiveness in colorectal cancer cell-lines [23, 24]. Our results confirm this observation and show that FMNL2 expression is up-regulated at both the mRNA and protein level in SW620 CRC cells when compared to their poorly invasive SW480 progenitor cell-line. Indeed, the level of total FMNL2 expression in SW620 cells is similar to that observed in the melanoma cell-lines. Surprisingly, FMNL2 is expressed to comparable levels in non-transformed primary melanocytes. As part of their normal physiological role melanocytes themselves are somewhat “invasive” and deliver melanosomes via widespread formation of filopodia [44–46]. Given the ability of FMNL2 to support invasion and induce filopodia formation, it might therefore be expected that elevated levels of FMNL2 expression would also be required in these cells. A key difference between the primary melanocytes and melanoma cells, however, is the variety of FMNL2 isoforms expressed. As noted, the TQS isoform predominates in A375 and WM266.4 cells while at least four FMNL2 isoforms are expressed in primary melanocytes. This suggests an intriguing model where it is not the total level of FMNL2 expression that determines invasiveness in melanoma, but the specific isoform that is expressed.

The ITM, YHY, PMR and TQS isoforms of FMNL2 differ only at a relatively small region in their C-termini. Nevertheless, the sequences affected are suggested to be critical to the normal regulation and function of diaphanous-related formins (DRFs); the domain C-terminal to DAD is thought to influence both FH2 activity [42] and autoregulation [9, 42]. Consistent with this, we find that auto-inhibition of PMR is less efficient than the other three isoforms and that TQS is the isoform most efficient at promoting invasion. Beyond direct effects on FMNL2 activity, the DRF C-terminus may also serve as a target for multiple regulatory and accessory factors [10]. Indeed, a recent study investigating the role of FMNL2 in integrin trafficking found that the C-terminal tail of FMNL2.ITM is a target of PKCα-dependent phosphorylation [28]. A comparison of the four FMNL2 isoforms on the Eukaryotic Linear Motif resource (http://elm.eu.org/index.html) reveals that the PKCα site is conserved in the ITM and YHY isoforms, but not in PMR or TQS. Instead the TQS C-terminal tail contains consensus PKB and proline-directed kinase sites and PMR contains no conserved kinase consensus sites at all. This comparison suggests that the C-terminal tail of each isoform is likely to be targeted by a variety of differing upstream signaling pathways. Determining how the prevalence of each isoform affects the integration of specific signaling pathways that regulate the effects of FMNL2 on cell behavior will be of great future interest.

## Conclusions

We show here that FMNL2 expression is required for invasion in A375 and WM266.4 melanoma cells and, taken with previous results, this suggests that FMNL2 is likely to be generally required by melanoma cells for invasion. At least four alternatively-spliced FMNL2 isoforms are expressed in primary melanocytes as well as in specific cancer cell-lines and sequence analysis of the encoded proteins suggests they will be targeted by distinct regulatory pathways. Of the four isoforms, FMNL2.TQS is preferentially up-regulated in invasive CRC and melanoma cell-lines and is the most efficient in supporting cellular invasion. This raises the possibility that TQS could be considered as an invasive isoform of FMNL2, analogous to Mena^INV^ and the invasive isoform of palladin. Regardless, our results suggest that future studies on the role of FMNL2 in metastasis should concentrate on TQS as the most relevant isoform.

## Abbreviations

CRC: Colorectal cancer
DAD: Diaphanous Autoregulatory domain
DID: Diaphanous inhibitory domain
DRF: Diaphanous-Related Formin
FH: Formin homology
FMNL: Formin-like
MT: Microtubules
qPCR: quantitative PCR
RT-PCR: Reverse transcription PCR
SRF: Serum Response Factor

## References

[1] Nurnberg A, Kitzing T, Grosse R (2011). Nucleating actin for invasion. Nat Rev Cancer.

[2] Petrie RJ, Yamada KM (2012). At the leading edge of three-dimensional cell migration. J Cell Sci.

[3] Sahai E, Marshall CJ (2003). Differing modes of tumour cell invasion have distinct requirements for Rho/ROCK signalling and extracellular proteolysis. Nat Cell Biol.

[4] Friedl P, Wolf K (2003). Tumour-cell invasion and migration: diversity and escape mechanisms. Nat Rev Cancer.

[5] Sanz-Moreno V, Marshall CJ (2010). The plasticity of cytoskeletal dynamics underlying neoplastic cell migration. Curr Opin Cell Biol.

[6] Kitzing TM, Wang Y, Pertz O, Copeland JW, Grosse R (2010). Formin-like 2 drives amoeboid invasive cell motility downstream of RhoC. Oncogene.

[7] Faix J, Grosse R (2006). Staying in shape with formins. Dev Cell.

[8] Paul AS, Pollard TD (2009). Review of the mechanism of processive actin filament elongation by formins. Cell Motil Cytoskeleton.

[9] Schonichen A, Alexander M, Gasteier JE, Cuesta FE, Fackler OT (2006). Biochemical characterization of the diaphanous autoregulatory interaction in the formin homology protein FHOD1. J Biol Chem.

[10] Chesarone MA, DuPage AG, Goode BL (2010). Unleashing formins to remodel the actin and microtubule cytoskeletons. Nat Rev Mol Cell Biol.

[11] Vaillant DC, Copeland SJ, Davis C, Thurston SF, Abdennur N (2008). Interaction of the N- and C-terminal autoregulatory domains of FRL2 does not inhibit FRL2 activity. J Biol Chem.

[12] Thurston SF, Kulacz WA, Shaikh S, Lee JM, Copeland JW (2012). The ability to induce microtubule acetylation is a general feature of formin proteins. PLoS One.

[13] Bartolini F, Moseley JB, Schmoranzer J, Cassimeris L, Goode BL (2008). The formin mDia2 stabilizes microtubules independently of its actin nucleation activity. J Cell Biol.

[14] Bartolini F, Gundersen GG (2010). Formins and microtubules. Biochim Biophys Acta.

[15] Favaro P, Traina F, Machado-Neto JA, Lazarini M, Lopes MR (2013). FMNL1 promotes proliferation and migration of leukemia cells. J Leukoc Biol.

[16] Favaro PM, de Souza Medina S, Traina F, Basseres DS, Costa FF (2003). Human leukocyte formin: a novel protein expressed in lymphoid malignancies and associated with Akt. Biochem Biophys Res Commun.

[17] Favaro PM, Traina F, Vassallo J, Brousset P, Delsol G (2006). High expression of FMNL1 protein in T non-Hodgkin’s lymphomas. Leuk Res.

[18] Martin-Rufian M, Segura JA, Lobo C, Mates JM, Marquez J (2006). Identification of genes downregulated in tumor cells expressing antisense glutaminase mRNA by differential display. Cancer Biol Ther.

[19] Vega FM, Fruhwirth G, Ng T, Ridley AJ (2011). RhoA and RhoC have distinct roles in migration and invasion by acting through different targets. J Cell Biol.

[20] Lynch J, Meehan MH, Crean J, Copeland J, Stallings RL (2013). Metastasis suppressor microRNA-335 targets the formin family of actin nucleators. PLoS One.

[21] Zeng YF, Xiao YS, Lu MZ, Luo XJ, Hu GZ (2015). Increased expression of formin-like 3 contributes to metastasis and poor prognosis in colorectal carcinoma. Exp Mol Pathol.

[22] Li Y, Zhu X, Zeng Y, Wang J, Zhang X (2010). FMNL2 enhances invasion of colorectal carcinoma by inducing epithelial-mesenchymal transition. Mol Cancer Res.

[23] Zhu XL, Liang L, Ding YQ (2008). Overexpression of FMNL2 is closely related to metastasis of colorectal cancer. Int J Color Dis.

[24] Zhu XL, Zeng YF, Guan J, Li YF, Deng YJ (2011). FMNL2 is a positive regulator of cell motility and metastasis in colorectal carcinoma. J Pathol.

[25] Ross DT, Scherf U, Eisen MB, Perou CM, Rees C (2000). Systematic variation in gene expression patterns in human cancer cell lines. Nat Genet.

[26] Block J, Breitsprecher D, Kuhn S, Winterhoff M, Kage F (2012). FMNL2 drives actin-based protrusion and migration downstream of Cdc42. Curr Biol.

[27] Grikscheit K, Frank T, Wang Y, Grosse R (2015). Junctional actin assembly is mediated by Formin-like 2 downstream of Rac1. J Cell Biol.

[28] Wang Y, Arjonen A, Pouwels J, Ta H, Pausch P (2015). Formin-like 2 Promotes beta1-Integrin Trafficking and Invasive Motility Downstream of PKCalpha. Dev Cell.

[29] Moriya K, Yamamoto T, Takamitsu E, Matsunaga Y, Kimoto M (2012). Protein N-myristoylation is required for cellular morphological changes induced by two formin family proteins, FMNL2 and FMNL3. Biosci Biotechnol Biochem.

[30] Gasman S, Kalaidzidis Y, Zerial M (2003). RhoD regulates endosome dynamics through Diaphanous-related Formin and Src tyrosine kinase. Nat Cell Biol.

[31] Iskratsch T, Lange S, Dwyer J, Kho AL, dos Remedios C (2010). Formin follows function: a muscle-specific isoform of FHOD3 is regulated by CK2 phosphorylation and promotes myofibril maintenance. J Cell Biol.

[32] Ramabhadran V, Korobova F, Rahme GJ, Higgs HN (2011). Splice variant-specific cellular function of the formin INF2 in maintenance of Golgi architecture. Mol Biol Cell.

[33] Han Y, Eppinger E, Schuster IG, Weigand LU, Liang X (2009). Formin-like 1 (FMNL1) is regulated by N-terminal myristoylation and induces polarized membrane blebbing. J Biol Chem.

[34] Kobielak A, Pasolli HA, Fuchs E (2004). Mammalian formin-1 participates in adherens junctions and polymerization of linear actin cables. Nat Cell Biol.

[35] Goicoechea SM, Bednarski B, Stack C, Cowan DW, Volmar K (2010). Isoform-specific upregulation of palladin in human and murine pancreas tumors. PLoS One.

[36] Goicoechea SM, Garcia-Mata R, Staub J, Valdivia A, Sharek L (2014). Palladin promotes invasion of pancreatic cancer cells by enhancing invadopodia formation in cancer-associated fibroblasts. Oncogene.

[37] Di Modugno F, Iapicca P, Boudreau A, Mottolese M, Terrenato I (2012). Splicing program of human MENA produces a previously undescribed isoform associated with invasive, mesenchymal-like breast tumors. Proc Natl Acad Sci U S A.

[38] Goswami S, Philippar U, Sun D, Patsialou A, Avraham J (2009). Identification of invasion specific splice variants of the cytoskeletal protein Mena present in mammary tumor cells during invasion in vivo. Clin Exp Metastasis.

[39] Copeland JW, Treisman R (2002). The diaphanous-related formin mDia1 controls serum response factor activity through its effects on actin polymerization. Mol Biol Cell.

[40] Young KG, Thurston SF, Copeland S, Smallwood C, Copeland JW (2008). INF1 is a novel microtubule-associated formin. Mol Biol Cell.

[41] Hetheridge C, Scott AN, Swain RK, Copeland JW, Higgs HN (2012). The formin FMNL3 is a cytoskeletal regulator of angiogenesis. J Cell Sci.

[42] Gould CJ, Maiti S, Michelot A, Graziano BR, Blanchoin L (2011). The formin DAD domain plays dual roles in autoinhibition and actin nucleation. Curr Biol.

[43] Philippar U, Roussos ET, Oser M, Yamaguchi H, Kim HD (2008). A Mena invasion isoform potentiates EGF-induced carcinoma cell invasion and metastasis. Dev Cell.

[44] Beaumont KA, Hamilton NA, Moores MT, Brown DL, Ohbayashi N (2011). The recycling endosome protein Rab17 regulates melanocytic filopodia formation and melanosome trafficking. Traffic.

[45] Yamaguchi Y, Hearing VJ (2014). Melanocytes and their diseases. Cold Spring Harb Perspect Med.

[46] Scott G, Leopardi S, Printup S, Madden BC (2002). Filopodia are conduits for melanosome transfer to keratinocytes. J Cell Sci.

